# Electrocardiographic Changes and Serum Troponin Levels in Patients with Acute Stroke: A Prospective Cross-Sectional Study

**DOI:** 10.4314/ejhs.v32i3.10

**Published:** 2022-05

**Authors:** Etedal Ahmed A Ibrahim, Salma Mohmed Taha, Khabab Abbasher Hussien Mohamed Ahmed, Mazin S Haroun, Mohammed Eltahier Abdalla Omer

**Affiliations:** 1 MBBS, MD, DCN (London), FRCP (London), Consultant Neurologist, Al Neelain University, Faculty of Medicine, Khartoum, Sudan; 2 MBBS, MD Internal medicine, The National center for Neurological Science, Khartoum, Sudan; 3 University of Khartoum, Faculty of Medicine, Khartoum, Sudan; 4 University of Khartoum, Faculty of Medicine, Khartoum, Sudan; 5 University of Gadarif, Faculty of Medicine, Gadarif, Sudan

**Keywords:** ECG, Stroke, Troponin, Neurology, Sudan

## Abstract

**Background:**

Electrocardiographic changes and elevated serum troponin are frequent findings in acute stroke. They may reflect what is known as the neurogenic myocardial injury. The aim of this study is to determine the electrocardiographic changes and serum troponin level in acute stroke patients and to correlate these changes to the anatomical location and pathological type of the stroke.

**Methods:**

A prospective cross-sectional study was conducted at the National Center of Neurological Science, from January to December 2019. Non-probability sampling with total coverage was considered. 50 patients with acute stroke were included in the study. Data were analyzed by using (SPSS) version 25. Standardized ECG was performed in the first hours of admission. 2 samples from each patient were obtained for serum troponin with at least 8 hours apart.

**Results:**

All patients had wide variants of ECG changes. But tachycardia was the most frequent one identified in 54% of patients (n=50). Half of them were found to have an anterior circulation stroke. 14% of patients (n=50) have positive troponin; ECG changes are identified in all patients who represent positive troponin 100% (7 patients). Moreover, anterior circulation stroke was recognized in all patients with a positive troponin I marker.

**Conclusion:**

This study suggests that ECG abnormalities in patients with acute stroke are very common, especially tachycardia. The site of the lesion appears to play a major factor as a cause of the genesis of arrhythmia. Serum troponin elevation may play a role in diagnosing neurocardiogenic injury; nevertheless, ECG appears to be more sensitive and familial.

## Introduction

Cardiovascular abnormalities are common following strokes. Disorders of the central nervous system cause a wide array of cardiovascular system dysfunctions, ranging from electrocardiogram changes and transient myocardial dysfunction to sudden death (1). Cardiovascular effects of stroke are modulated by concomitant or preexistent cardiac disease and may be related to the pathological type of cerebrovascular disease and its localization (2). It is essential to distinguish whether cardiovascular abnormalities are caused by stroke, unrelated to it, or in the course of cardiac arrhythmia, that leads to stroke. The distinction is often difficult because preexisting cardiac abnormalities are highly prevalent among stroke (3).

There is considerable clinical and experimental evidence that stimulation of certain areas in the brain such as the insular cortex can induce cardiac dysfunction in the form of myocytolysis, enzyme elevation and arrhythmias (4). Furthermore, increased sympathetic activity is thought to be contributory, inducing reversible cardiac myocardium damage through catecholamine & cortisol surge (5).

Early detection and management of cardiac complications post stroke may carry prognostic value, as cardiac abnormalities can greatly increase the morbidity and mortality of patients with intracranial pathologies (6). Stroke brings high morbidity and mortality rates; it is a major health problem worldwide. An estimated 17.5 million people died from cerebrovascular diseases (CVDs) in 2012 representing 31% of all global deaths. An estimated 6.7 million were due to stroke (7). In Europe it is the second mortality cause, accounting for 10% of all deaths in men and 15% in women (8). Several studies have demonstrated that cardiac dysfunction may occur after vascular brain injuries without any evidence of primary heart disease. Autopsy showed subendothelial hemorrhage and ischemic changes that are not related to coronary artery distribution. In addition, autonomic dysfunction is common after acute stroke. This is evidenced by impairment of physiological regulation of heart rate and blood pressure, namely decreased heart rate variability (HRV), and impaired baroreceptors (9). The variability in heart rate is indicative of the heart's ability to adjust to circulatory changes. Several studies have reported decreased HRV in patients with stroke, not only in the acute phase but also at 1 to 6 months after stroke.

Autonomic dysfunction causing increased surge of catecholamine and cortisol. Catecholamine excess over activates the B adrenergic receptors, over activation of these receptors leads to tonic opening of Ca channels causing impairment in sequestration of intracellular Ca, which is necessary for relaxation of cardiac muscles. Sequestration of intracellular Ca prolongs repolarization period and hence, prolonged contraction of cardiac muscle which leads to cell damage and death (9).

Elevated cardiac markers after stroke support the autonomic imbalance theory but do not necessarily indicate that myocardial injury has occurred. In a study CK, CK-MB, myoglobins, were found to be elevated, following right hemispheric stimulation, 3 days after stroke and returning to normal in the 4th day (11). Troponin T, which is more specific for myocardial injury, was in the reference range. In another study conducted in 730 patients with acute stroke, without known heart or renal disease and no clinical ECG or echocardiography changes suggestive of coronary artery diseases, found that 7% of patients had elevated troponin levels. A recent study mentioned the level of cardiac troponin in patients with stroke and the pattern of acute or chronic elevation with clinical conditions can help to classify the coronary causes and neurogenic heart syndrome morbidity and mortality after acute stroke at 30 days to 6 months. However, the clinical significance of elevated cardiac bio -markers after acute stroke is inconclusive till now (9).

Anatomically insular cortex is an interconnecting part of the cerebral cortex located within the lateral sulcus of the brain. Stimulation of the insular cortex results in cardiovascular effects with some lateralization. Stimulation of the right insular cortex more frequently produces a sympathetic effect. Stimulation of the left cortical insula produces-parasympathetic effects (13). Small case reports of isolated insular lesions have suggested that right insular hemorrhagic or ischemic strokes resulted in bradycardia and sometimes asystole. This can be explained by the decreased sympathetic tone from the right insular lesion and relative parasympathetic overactivity. On the contrary, isolated left insular lesions result in decreased HRV possibly due to loss of parasympathetic tone and increased basal effects of sympathetic cardiac activity. However, assuming that there is complete lateralization of autonomic control from the insular cortex is inappropriate as the literature reports conflicting results on this topic, some suggest lateralization where others merge against this theory (10,12).

Our aim was to determine electrocardiographic changes and serum troponin level in patients with acute stroke and to correlate these changes to the anatomical location and pathological type of the stroke.

## Methods

This was a prospective cross-sectional study. It was conducted at the national center for neurological science, Khartoum Sudan during the period from February 2019 to December 2019. All cases presented with acute stroke during the study period were included. National center for Neurological Science is considered to be one of the main reference teaching hospitals in Sudan. In this center there is a main laboratory, X-ray department, pharmacy, physiotherapy department and ICU. The center has two general wards (medical and surgical) with a capacity of 110 beds, 24 of them in the medical ward. The medical ward receives about 670 patients per year. There are four outpatient clinics which receive about 3000 patients per year referred from all over Sudan. A non- probability sampling technique was used, with total coverage during the mentioned study period. A total of 50 patients were enrolled in the study.

**Inclusion criteria**

Sudanese patients above 16 years old presenting with acute stroke within the first 72 hours. Brain imaging, electrocardiography and serum troponin level were mandatorily investigated in all included patients.

**Exclusion criteria**

Patients below 16 years old.

Hemiplegia due to other neurological problems.

Psychological hemiplegia.

Patients with chronic kidney disease, chronic obstructive pulmonary diseases, or congestive cardiac failure.

The patients of acute stroke were identified, and the well-structured questionnaire was filled by a consultant physician during the first 72 hours: A well-constructed pretested questionnaire which includes demographic data, past medical history, examination findings, imaging study, electrocardiographic records and troponin status. Data were analyzed by using a computer program statistical package of social science (SPSS) version 25. Ethical considerations, including verbal consent were obtained from patients/copatients after explanation of the study, its nature, the confidentiality of the data and their right to quit the study at any time during the study. Ethical approval was obtained from the Sudan specialization board ethical committee and the National Center for neurological science's ethical committee.

## Results

A total of 50 patients with acute stroke within 72 hours of symptom onset were included in the study. ECG was performed on all patients during the first hours of admission; brain imaging and serum troponin I level were studied. The mean age was 70.3+/-6.4 SD. About 26 (52%) of patients were males, and 24 (48%) were females (n=50). Regarding the risk factors, hypertension was found in 86% of the patients (n=50). About 11 patients (22%) are known diabetics. Hyperlipidemia was identified in 10%. Moreover, 10% mentioned the previous history of transient Ischemic attack (TIA). Overall 13 patients (26%) admitted to being smokers, whereas 3 patients (6%) consumed alcohol (n=50). The patients who have had chronic atrial fibrillation constitute 6% (3 patients). Almost two patients (4%) are known to have ischemic heart disease, two patients (4%) are diagnosed with valvular lesions and 6 patients (12%) have no obvious risk factors.

Using the Rosier scale the suspected stroke patients were 48/50. 2 patients with scores less than 0 were confirmed to have a stroke by imaging.

Score 2 Rosier stroke scale was the most frequent, documented in 22 patients (44%). 15 patients scored 3 (30%). Confirmatory imaging was done for 41 patients (82%). CT-brain scan and MRI-brain were done for 9 patients (18%) respectively. Overall 50% (25) of the strokes were ischemic in nature. While 50% of patients (25), were hemorrhagic in nature. Subarachnoid hemorrhage was found in 2 patients.

Distribution of areas affected was as follows: The temporoparietal region was found to be the most affected area, 10 of these patients (20%) suffered from hemorrhagic stroke, while 9 patients (18%) suffered from ischemic stroke. Basal ganglia were the second most affected area in which 4 patients (8%) suffered from hemorrhagic stroke and 4 patients (8%) suffered from ischemic stroke. Third most affected area was the occipitoparietal area, 4 patients (8%) suffered from hemorrhagic stroke, and 3 patients (6%) suffered from ischemic stroke.

Other sites of strokes such as hypothalamic, parietal, occipital and temporal strokes were evident, in percentages ranging from 4% to 2%. 37 patients from this study (74%) suffered from a stroke involving the anterior circulation. Posterior circulation strokes were identified in 9 patients, which represented 18% of the sample size. Subarachnoid hemorrhage was found in 2 patients (4%), in addition, 2 patients had bilateral/multiple strokes (4%).

**Electrocardiographic Changes**: All ECG traces in this study showed variant abnormalities. The highest frequencies were observed for tachycardia found in 27 patients (54%), (13 of which had temporoparietal lesions). The second most common ECG finding was left ventricular hypertrophy seen in 24 patients (48%).

Bradycardia was evident in 20%, identified in 10 patients. Sharing the same percentage was patients with ST-segment depression. Other Ischemic changes observed are T wave depression in 5 patients (10%). Significant STsegment elevation was identified once. Arrhythmias, including supraventricular, were recorded, atrial fibrillation in 6 patients (12%), atrial flutter in 1 patient (2%), others such as small vessel disease and premature atrial contractions were recorded as well. Ventricular fibrillation was identified in one patient. Other abnormal presentations were RBBB, LBBB, abnormal Q Wave and prolonged QT wave.

**Troponin status**: Troponin I was positive in 7 patients (14%), whereas 43 patients (86%) were negative twice. In the positive troponin patients, anterior circulation stroke was identified (5 patients with temporoparietal stroke, 2 patients with basal ganglia stroke).

ECG changes are identified in all positive troponin patients. Supra ventricular arrhythmias were the most frequent. Ventricular fibrillation was recorded once.

## Discussion

We found that ECG abnormalities in patients with acute stroke are very common, especially tachycardia. The site of lesions appears to play a major factor as a cause of genesis of arrhythmia. Concomitant cardiac diseases may present. Serum troponin elevation may play a role in diagnosing neuro-cardiogenic injury but, ECG appears to be more sensitive and familial.

Sadber et. al mentioned that autonomic control was decreased in patients with stroke and a more pronounced decrease was found in the middle cerebral artery insular cortex (14). All traces in this study showed ECG abnormalities, the most frequent changes were tachycardia which was present in 27 patients (54%). Left ventricular hypertrophy features were found in 24/34 of the hypertensive patients. Bradycardia and ST depression share the same percentages of 20%. T wave inversion was observed at 10%. In contrast to (Ghola Merza B et.al) who mentioned ST depression and T inversion as the most frequent changes, the same goes for another study done by (Asadi P. et al) (18,19).

Our observation was similar to what was achieved by (Sullvin Lavy et al.) who found that both disturbances in rhythm, conduction and ischemic-T alteration were detected but frequency of the former exceeded that of later (15). Supra ventricular arrhythmias were caught; AF was the most frequent 12%, although half of them are known cases of chronic AT. This is followed by atrial flutter, SVT, PCA, also those findings are similar to findings of the Iranian study. In this study, fatal arrhythmia VF was seen once. Other ECG changes observed in small proportions are RBBB, LBBB, abnormal Q waves, and prolonged QT interval. Regarding rate changes and location; tachycardia was more frequently identified in the temporoparietal lesions. 14% affect the right temporoparietal lobe .12% affect the left. Bradycardia was a closed finding to occipital and occipito-parietal lesions, and was documented in subarachnoid hemorrhage. The relation of rhythm changes to stroke location has no statistical significance (p=0.9). A rare finding was the presence of bradycardia in left temporoparietal stroke; this may explain the dominant parasympathetic tone of the left insula. But lateralization showed no statistical significance in this study.

Troponin I is a sensitive marker of cardiac alteration, elevated in myocardial infarction, myocarditis, pericarditis, atrial fibrillation and heart failure. Elevated troponin I has also been found in patients with chronic renal failure, sepsis, critical illness, pulmonary embolism and COPD (16).

Elevated levels of troponin have been reported in 10–34% of patients with acute stroke (Kerr et al) (17). In this study 14 % have positive readings matching what have been reported. Trying to localize stroke in those with positive troponin (7 patients), it was clear that all of them had an anterior circulation stroke. (5/7) suffered a temporoparietal stroke. The other two patients presented with basal ganglia stroke. All of them showed ECG changes. Increased heart rate and supraventricular arrhythmias were recognized. Fatal ventricular fibrillation was identified in one patient. The Turkish study state that 5 patients with RMCA- insular lesions died suddenly compared with two patients of LMCA -insular lesions during hospitalization which suggests that cardiac autonomic tone may be regulated by insula, and that these patients are more prone to cardiac complications such as arrhythmias. In this study it is found that ST segment /T wave inversion is seen more with the right temporoparietal lesions than the left lesions.

To our knowledge this is the first study to be done in Sudan concerning this topic. We emphasized on being strict with the inclusion and exclusion criteria of selecting proper candidates to enter the study to ensure the highest possible accuracy.

First of all, the sample size was not as anticipated because in patients with stroke, the condition of patients is usually critical, therefore it wasn't easy to collect more samples. Also, the number of the doctors was not sufficient to collect more samples, as many of the doctors were occupied with the events relating to the Sudanese revolution at that time. In addition, it was not feasible to perform many investigations, nor investigate all the patients included in the sample due to the lack of proper facilities and available infrastructure. Also, we couldn't be able to add a control group of healthy individuals due to the shortage of resources and absence of funding.

The mechanism explaining morphological electrocardiogram (ECG) changes and increase in troponin in acute stroke is not clear. The observation of this study suggests ECG abnormalities in patients with acute stroke are very common. Concomitant cardiac diseases may present. The site of the lesion appears as a factor of the genesis of arrhythmia. Serum troponin elevation may play a role in the diagnosis of neurogenic injury, plus ECG which appears to be non-costive.

ECG changes are justified for intensive monitoring. Locating the stroke may reflect future cardiac dysfunction. Identifying preexisting cardiac disease is important. Advanced facilities such as echocardiography are needed at the level of causality/Emergency room. Improving stroke care capabilities may improve stroke outcomes.
